# Detection of *bla*_OXA-23_–Positive *Proteus mirabilis* Isolate, United States, 2024

**DOI:** 10.3201/eid3208.260588

**Published:** 2026-08

**Authors:** Giulia Orazi, Porscha Bumpus-White, Alyssa G. Kent, Erin Breaker, Matthew Doucette, Caryn Ivanof, Esther Fortes, Sanjib Bhattacharyya, Nicolas Epie, Susannah L. McKay, Stephen P. LaVoie, Sarah Sabour

**Affiliations:** Centers for Disease Control and Prevention, Atlanta, Georgia, USA (G. Orazi, P. Bumpus-White, A.G. Kent, E. Breaker, S.L. McKay, S.P. LaVoie, S. Sabour); Massachusetts State Public Health Laboratory, Department of Public Health, Jamaica Plain, Massachusetts, USA (M. Doucette, C. Ivanof, E. Fortes, S. Bhattacharyya, N. Epie)

**Keywords:** Proteus mirabilis, bacteria, antimicrobial resistance, carbapenem, class D carbapenemase gene, Enterobacterales, *Acinetobacter baumannii*, Antimicrobial Resistance Laboratory Network, United States

## Abstract

We report detection of a *bla*_OXA-23_-positive *Proteus mirabilis* clinical isolate through Antimicrobial Resistance Laboratory Network testing that is closely related to OXA-23 producers identified in Europe. Combined with similar reports internationally, this finding suggests that Enterobacterales might serve as a silent reservoir of carbapenemase genes commonly associated with *Acinetobacter* species.

The Ambler class D β-lactamase OXA-23–like subgroup is among the most common acquired mechanisms contributing to carbapenem resistance in *Acinetobacter baumannii* in the United States and globally (*1*). Detection of *bla*_OXA-23_ has been infrequently reported outside of the genus *Acinetobacter*; *bla*_OXA-23_–positive isolates of *Proteus mirabilis* are increasingly reported in Europe, particularly in France (*2*–*7*). Through routine testing conducted by the Centers for Disease Control and Prevention (CDC) Antimicrobial Resistance Laboratory Network (AR Lab Network), we detected a *P. mirabilis* clinical isolate from Massachusetts, USA, harboring *bla*_OXA-23_. This activity was reviewed and approved by CDC as exempt human subjects research (see 45 C.F.R. part 46.104).

*P. mirabilis* was isolated in May 2024 from a urine culture of a patient receiving outpatient care in Massachusetts. In the 12 months before specimen collection, the patient had no reported history of international travel, domestic inpatient healthcare stays, or invasive procedures. As part of the AR Lab Network, clinical laboratories submit clinical isolates of carbapenem-resistant Enterobacterales to public health laboratories for characterization, as previously described (*1*) (Appendix).

We performed supplementary antimicrobial susceptibility testing at CDC by reference broth microdilution according to Clinical Laboratory Standards Institute (CLSI) guidelines as previously described (*8*); we interpreted results using CLSI breakpoints (*9*). The isolate was susceptible to both ertapenem and meropenem by broth microdilution and disk diffusion (Table), consistent with the characteristically weak carbapenemase activity of OXA-23 (*2*). The isolate was susceptible to all other β-lactams except ampicillin and doripenem (and also imipenem, to which *Proteus* spp. commonly have reduced susceptibility) (*9*) and all tested cephalosporins apart from cefazolin (Table). Of note, the isolate showed susceptibility to piperacillin/tazobactam (Table), which is less frequently observed for *bla*_OXA-23_–positive isolates (*6*). The isolate was positive for carbapenemase production but PCR-negative for the 5 carbapenemase genes targeted by the AR Lab Network.

**Table:** Antimicrobial susceptibility testing profile of a *bla*_OXA-23_-positive *Proteus mirabilis* isolate (2024DK-00154) detected through the Centers for Disease Control and Prevention Antimicrobial Resistance Laboratory Network, Massachusetts, USA, 2024*

Antimicrobial class	Antimicrobial	Broth microdilution			Disk diffusion	
		MIC, µg/mL	Interpretation		Zone size, mm	Interpretation
Carbapenems	Ertapenem	<0.12	S		25	S
	Meropenem	0.5	S		23	S
	Imipenem†	4	R		22	I
	Doripenem	NT	NA		22	I
Cephems (Cephalosporins)	Cefazolin	NT	NA		18	R
	Cefepime	<0.5	S		30	S
	Cefotaxime	<0.5	S		NT	NA
	Cefoxitin	<2	S		NT	NA
	Ceftazidime	<1	S		31	S
	Ceftriaxone	<1	S		35	S
	Cefiderocol	<0.03	S		NT	NA
Monobactams	Aztreonam	<1	S		36	S
β-lactam combination agents	Ceftazidime/avibactam	<0.5/4	S		NT	NA
	Ceftolozane/tazobactam	<0.5/4	S		NT	NA
	Meropenem/vaborbactam	<0.5/8	S		NT	NA
	Piperacillin/tazobactam	<4/4	S		22	SDD
Penicillins	Ampicillin	>32	R		NT	NA
Folate pathway antagonists	Trimethoprim/sulfamethoxazole	>8/152	R		NT	NA
Fluoroquinolones	Ciprofloxacin	<0.25	S		NT	NA
	Levofloxacin	<0.12	S		NT	NA
Aminoglycosides	Amikacin	<1	S		NT	NA
	Gentamicin	>16	R		NT	NA
	Tobramycin	16	R		NT	NA

*MICs were obtained by reference broth microdilution performed at Centers for Disease Control and Prevention according to Clinical and Laboratory Standards Institute guidelines. Kirby-Bauer disk diffusion was performed by the Massachusetts public health laboratory. Results were interpreted according to Clinical and Laboratory Standards Institute guidelines (*9*). I, intermediate; NA, not applicable; NT, not tested; R, resistant; S, susceptible; SDD, susceptible–dose dependent. †*P. mirabilis* isolates commonly exhibit elevated MICs to imipenem by mechanisms other than carbapenemase production.

We performed whole-genome sequencing (WGS) and data analysis; we submitted WGS data for the strain, 2024DK-00154, to the National Center for Biotechnology Information BioSample database (https://www.ncbi.nlm.nih.gov/biosample; accession no. SAMN41612253) (Appendix). We obtained a closed genome of 3.93 Mb that was not found to harbor any plasmids. The β-lactamase gene *bla*_OXA-23_ and 9 other AR genes were detected in this isolate (*tetJ*, *aadA1*, *dfrA1*, *sat2*, *sul2*, *aph(*6*)-Id*, *aph(3”)-Ib*, *aac(*3*)-IIe*, *aph(3′)-Ia*). The *bla*_OXA-23_ gene is located inside a Tn2008-like transposon (Tn6704) within a Tn6703-like genomic island, closely resembling the *bla*_OXA-23_ region of strains from Switzerland (Pm1 and Pm5) (*7*) and France (VAC) (*4*) (Figure, panel A).

**Figure:**
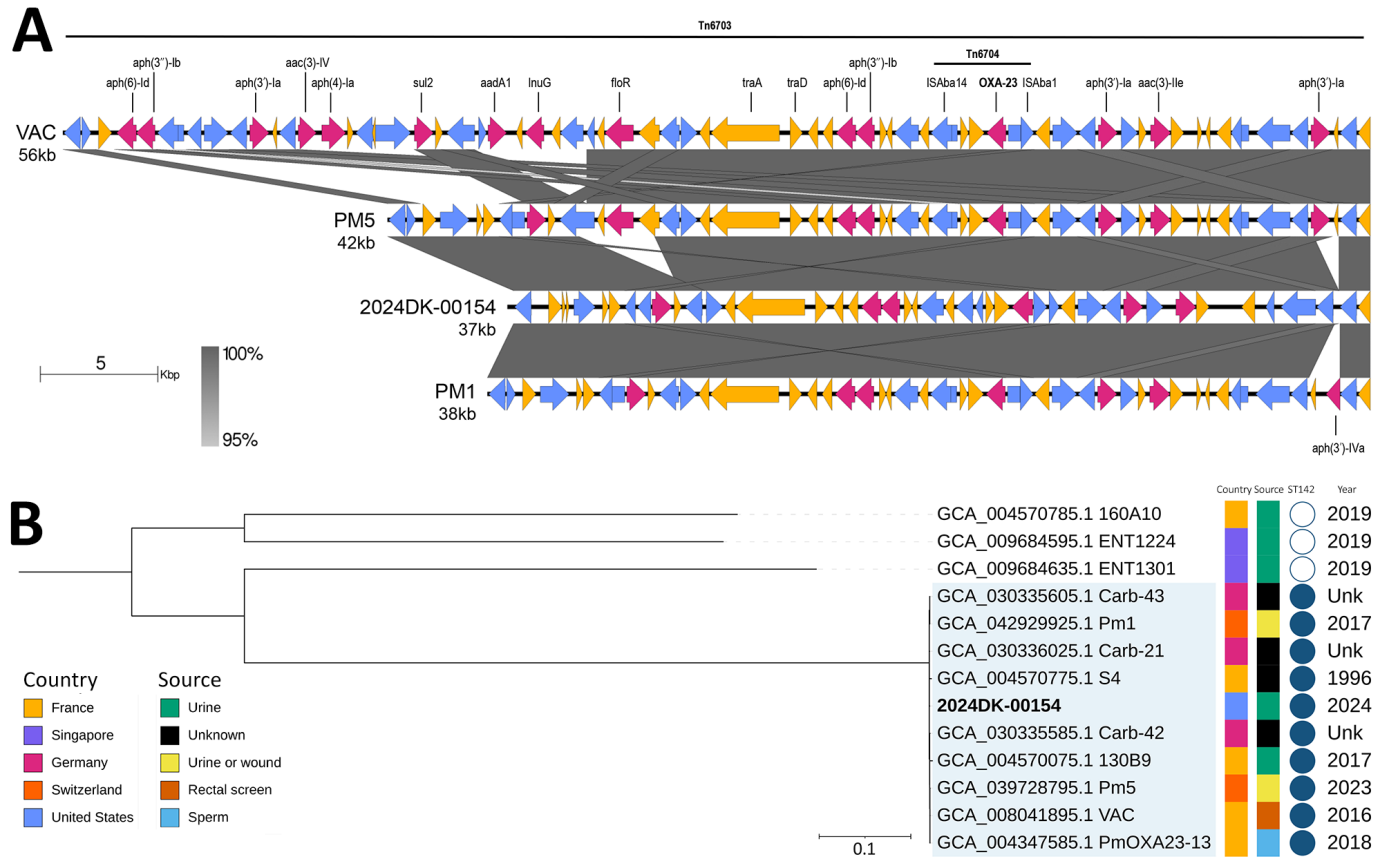
Genomic comparisons of *bla*_OXA-23_–positive *Proteus mirabilis* detected in Massachusetts, USA, in 2024 to clinical isolates identified in previous studies. A) Genomic context of *bla*_OXA-23_ regions of *P. mirabilis* isolate detected in Massachusetts (2024DK-00154) and *P. mirabilis* clinical isolates previously identified in Switzerland (Pm1 and Pm5) and France (VAC). BLASTn (https://blast.ncbi.nlm.nih.gov) was used to identify the best match to the National Center for Biotechnology Information nucleotide database (https://www.ncbi.nlm.nih.gov/nucleotide) of the *bla*_OXA-23_-containing region. Genomic region comparison was generated using Easyfig (https://mjsull.github.io/Easyfig). Pink indicates antimicrobial resistance genes; blue indicates insertion sequences and transposon genes; yellow indicates other genes. Grey shading represents percent nucleotide sequence identity as indicated by the key. B) Phylogenetic tree based on core-genome alignment of *bla*_OXA-23_–positive *P. mirabilis* isolate detected in Massachusetts (2024DK-00154, in bold; GenBank accession no. CP194042) and *bla*_OXA-23_–positive *P. mirabilis* clinical isolates previously identified in other countries (GenBank accession nos. GCA_004347585.1a, GCA_004570075.1, GCA_004570775.1, GCA_004570785.1, GCA_008041895.1, GCA_009684595.1, GCA_009684635.1, GCA_030335585.1, GCA_030335605.1, GCA_030336025.1, GCA_039728795.1, GCA_042929925.1; genome assembly sizes min: 3.91 Mb; max: 4.11 Mb; average: 4.00 Mb). Core-genome alignment and construction of a maximum-likelihood tree were performed using Parsnp (https://github.com/marbl/parsnp). Tree was visualized using iTOL (https://itol.embl.de) and rooted at the midpoint. Country and source of isolate collection are specified. Filled blue circles represent sequence type (ST) 142 isolates; empty circles represent non-ST142 isolates. Year of isolate collection is provided when known. Blue box indicates ST142 isolate cluster. Scale bar indicates number of substitutions per nucleotide. Unk, unknown.

We inferred a phylogenetic tree of 2024DK-00154 and 12 other *bla*_OXA-23_-positive *P. mirabilis* strains on the basis of the alignment of the core genome (≈3.23 Mb; 81% of the average genome size) (Figure, panel B). The 2024DK-00154 strain clustered with clinical isolates collected in Europe during 1996–2023, including an OXA-23–producing lineage circulating in France and Belgium since 1996 (*2*,*4*) and closely related strains detected in Germany (*6*) and Switzerland (*7*) (Figure, panel B). Within that cluster, all isolates belonged to sequence type (ST) 142 and differed by 4–200 core-genome single-nucleotide variants (SNVs); 2024DK-00154 is most similar to a strain isolated in Germany (Carb-21), differing by only 27 SNVs across ≈80% of the genome. In contrast, 2024DK-00154 differs from non-ST142 isolates from Singapore and France in which *bla*_OXA-23_ is plasmidborne (*4*,*10*) by 17,321–18,347 SNVs.

Those results suggest that the *P. mirabilis* isolate identified in Massachusetts is closely related to contemporary *bla*_OXA-23_–positive ST142 *P. mirabilis* strains circulating in Europe, consistent with international dissemination. In France, OXA-23–producing *P. mirabilis* strains (that cluster with the Massachusetts isolate) have been recovered from epidemiologically unrelated patients, suggesting community spread (*3*). Community spread has not been detected in the United States but is possible. Indeed, the Massachusetts case-patient had no reported international travel, suggesting a local infection source.

Testing for carbapenemase genes in the AR Lab Network is initiated on the basis of the detection of carbapenem resistance at the clinical laboratory. Thus, *bla*_OXA-23_–positive *P. mirabilis* isolates, which are characteristically carbapenem-susceptible, would not be targeted for mechanism testing, and the presence of *bla*_OXA-23_ would go undetected. Consequently, appropriate infection control measures that would be taken for patients identified as harboring carbapenemase-producing organisms would not necessarily be taken; *P. mirabilis* could serve as an unmonitored reservoir enabling ongoing transmission of *bla*_OXA-23_ to other species, including other Enterobacterales species. The inability to detect reservoirs of carbapenemase genes, including environmental sources, healthcare workers, or novel gene-organism combinations, could pose challenges for identifying and addressing sources of transmission.

In addition to displaying susceptibility to carbapenems, *bla*_OXA-23_–harboring *P. mirabilis* might also escape detection during routine laboratory testing for carbapenemases because of poor sensitivity of phenotypic carbapenemase production tests and the absence of that target from most commercial PCR and immunochromatographic tests (*6*). A diagnostic algorithm based on antimicrobial susceptibility profiles and phenotypic assays was recently developed to improve detection of OXA-23 and other carbapenemases whose frequency might be underestimated in *Proteus* spp. (*3*,*4*,*6*). Our findings suggest the need for continued vigilance in detecting *bla*_OXA-23_ in genera beyond *Acinetobacter* to prevent further spread between taxa.

Appendix Additional information about detection of *bla*_OXA-23_–positive *Proteus mirabilis* isolate, United States, 2024.
