# Supplementary material for: Detection of blaOXA-23–Positive Proteus mirabilis Isolate, United States, 2024
Source: Emerg Infect Dis. 2026 Aug;32(8):1379–82. doi: 10.3201/eid3208.260588 (PMC13426824; doi:10.3201/eid3208.260588)
Supplement: Appendix — Additional information about detection of blaOXA-23–positive Proteus mirabilis isolate, United States, 2024. [file 26-0588-Techapp-s1.pdf]

Article DOI: https://doi.org/10.3201/eid3208.260588

EID cannot ensure accessibility for supplementary materials supplied by authors. Readers who have difficulty accessing supplementary content should contact the authors for assistance.

Detection of *bla*$_{OXA-23}$–Positive *Proteus mirabilis* Isolate, United States, 2024

Appendix

Supplementary Methods

AR Lab Network Testing

Public health laboratories (PHLs) in the Centers for Disease Control and Prevention Antimicrobial Resistance Laboratory Network perform routine testing including antimicrobial susceptibility testing (AST), phenotypic carbapenemase production testing, and real-time-PCR (RT-PCR) for detection of five targeted carbapenemase genes (*bla*$_{KPC}$, *bla*$_{NDM}$, *bla*$_{OXA-48-like}$, *bla*$_{IMP}$, and *bla*$_{VIM}$), as previously described (*1*,*2*). Whole-genome sequencing (WGS) is performed to characterize a subset of isolates, including those with discordant carbapenemase production and RT-PCR results (i.e., carbapenemase production-positive but RT-PCR-negative for the five targeted carbapenemase genes), which may indicate the presence of novel carbapenemase genes. The Massachusetts PHL performed AST by Kirby-Bauer disk diffusion and phenotypic carbapenemase production testing by the modified carbapenemase inactivation method.

Genomic Analyses

Short-read WGS was performed at the Massachusetts PHL on an Illumina MiSeq platform using the Nextera DNA Library Prep Kit. Raw reads were processed as previously described (*3*) and the *bla*$_{OXA-23}$ gene was identified using AMRFinderPlus (software v3.11.4; database v2023–02–23.1) (*4*). The CDC pipeline PHoeNIx v2.1.1 (https://github.com/CDCgov/phoenix) was used to process Illumina reads, perform genome assembly, and detect antimicrobial resistance genes (thresholds: 98% amino acid identity, 90%

coverage). Long-read WGS was performed at CDC using the Rapid Barcoding Kit V14 and an R10.4.1 flow cell on the MinION (Oxford Nanopore Technologies). Hybrid assembly was performed using Hybracter v0.11.0 (default settings hybrid-single with -auto) (*5*). BLASTn was used to identify the best match to the NCBI nucleotide database of the *bla*$_{OXA-23}$-containing region (*6*); alignment was generated using Easyfig (*7*). Phylogenetic tree was inferred based on alignment of the core genome performed using Parsnp v2.1.3 (default settings; reference strain: VAC [GCA_008041895.1]) (*8*). The tree was visualized using iTOL v7 (https://itol.embl.de/) (*9*) and rooted at the midpoint. WGS data were deposited to NCBI (BioSample: SAMN41612253; Sample name: 2024DK-00154; MinION reads: SRR33582377; Illumina reads: SRR29245601; Hybrid assembly: CP194042).

References

1. Sabour S, Bantle K, Bhatnagar A, Huang JY, Biggs A, Bodnar J, et al. Descriptive analysis of targeted carbapenemase genes and antibiotic susceptibility profiles among carbapenem-resistant *Acinetobacter baumannii* tested in the Antimicrobial Resistance Laboratory Network-United States, 2017–2020. Microbiol Spectr. 2024;12:e0282823. PubMed https://doi.org/10.1128/spectrum.02828-23

2. Sabour S, Huang JY, Bhatnagar A, Gilbert SE, Karlsson M, Lonsway D, et al. Detection and characterization of targeted carbapenem-resistant health care-associated threats: findings from the Antibiotic Resistance Laboratory Network, 2017 to 2019. Antimicrob Agents Chemother. 2021;65:e0110521. PubMed https://doi.org/10.1128/AAC.01105-21

3. Little JS, Coughlin C, Hsieh C, Lanza M, Huang WY, Kumar A, et al. Neuroinvasive *Bacillus cereus* infection in immunocompromised hosts: epidemiologic investigation of 5 patients with acute myeloid leukemia. Open Forum Infect Dis. 2024;11:ofae048. PubMed https://doi.org/10.1093/ofid/ofae048

4. Feldgarden M, Brover V, Gonzalez-Escalona N, Frye JG, Haendiges J, Haft DH, et al. AMRFinderPlus and the Reference Gene Catalog facilitate examination of the genomic links among antimicrobial resistance, stress response, and virulence. Sci Rep. 2021;11:12728. PubMed https://doi.org/10.1038/s41598-021-91456-0

5. Bouras G, Houtak G, Wick RR, Mallawaarachchi V, Roach MJ, Papudeshi B, et al. Hybracter: enabling scalable, automated, complete and accurate bacterial genome assemblies. Microb Genom. 2024;10:001244. PubMed https://doi.org/10.1099/mgen.0.001244

6. Altschul SF, Gish W, Miller W, Myers EW, Lipman DJ. Basic local alignment search tool. J Mol Biol. 1990;215:403–10. PubMed https://doi.org/10.1016/S0022-2836(05)80360-2

7. Sullivan MJ, Petty NK, Beatson SA. Easyfig: a genome comparison visualizer. Bioinformatics. 2011;27:1009–10. PubMed https://doi.org/10.1093/bioinformatics/btr039

8. Kille B, Nute MG, Huang V, Kim E, Phillippy AM, Treangen TJ. Parsnp 2.0: scalable core-genome alignment for massive microbial datasets. Bioinformatics. 2024;40:btae311. PubMed https://doi.org/10.1093/bioinformatics/btae311

9. Letunic I, Bork P. Interactive Tree of Life (iTOL) v6: recent updates to the phylogenetic tree display and annotation tool. Nucleic Acids Res. 2024;52(W1):W78–82. PubMed https://doi.org/10.1093/nar/gkae268
